# Meningococcal Disease in Children in Merseyside, England: A 31 Year Descriptive Study

**DOI:** 10.1371/journal.pone.0025957

**Published:** 2011-10-07

**Authors:** Michelle C. Stanton, David Taylor-Robinson, David Harris, Fauzia Paize, Nick Makwana, Scott J. Hackett, Paul B. Baines, F. Andrew I. Riordan, Omnia Marzouk, Alistair P. J. Thomson, Peter J. Diggle, C. Anthony Hart, Enitan D. Carrol

**Affiliations:** 1 Lancaster Medical School, Faculty of Health and Medicine, Lancaster University, Lancaster, United Kingdom; 2 Department of Health Inequalities and the Social Determinants of Health, Institute of Psychology, Health and Society, University of Liverpool, Liverpool, United Kingdom; 3 Department of Women's and Children's Health, Institute of Translational Medicine, University of Liverpool, Liverpool, United Kingdom; 4 Department of Child Health, Sandwell and West Birmingham NHS Trust, Lyndon, West Bromwich, West Midlands, United Kingdom; 5 Department of Paediatric Infectious Diseases and Immunology, Birmingham Heartlands Hospital, Bordesley Green East, Birmingham, United Kingdom; 6 Department of Professional Ethics, Keele University, Keele, United Kingdom; 7 Alder Hey Children's NHS Foundation Trust, Liverpool, United Kingdom; 8 Leighton Hospital, Crewe, Cheshire, United Kingdom; 9 Department of Clinical Infection, Microbiology and Immunology, Institute of Infection and Global Health, University of Liverpool, Liverpool, United Kingdom; Health Protection Agency, United Kingdom

## Abstract

Meningococcal disease (MCD) is the leading infectious cause of death in early childhood in the United Kingdom, making it a public health priority. MCD most commonly presents as meningococcal meningitis (MM), septicaemia (MS), or as a combination of the two syndromes (MM/MS). We describe the changing epidemiology and clinical presentation of MCD, and explore associations with socioeconomic status and other risk factors. A hospital-based study of children admitted to a tertiary children's centre, Alder Hey Children's Foundation Trust, with MCD, was undertaken between 1977 to 2007 (n = 1157). Demographics, clinical presentations, microbiological confirmation and measures of deprivation were described. The majority of cases occurred in the 1–4 year age group and there was a dramatic fall in serogroup C cases observed with the introduction of the meningococcal C conjugate (MCC) vaccine. The proportion of MS cases increased over the study period, from 11% in the first quarter to 35% in the final quarter. Presentation with MS (compared to MM) and serogroup C disease (compared to serogroup B) were demonstrated to be independent risk factors for mortality, with odds ratios of 3.5 (95% CI 1.18 to 10.08) and 2.18 (95% CI 1.26 to 3.80) respectively. Cases admitted to Alder Hey were from a relatively more deprived population (mean Townsend score 1.25, 95% CI 1.09 to 1.41) than the Merseyside reference population. Our findings represent one of the largest single-centre studies of MCD. The presentation of MS is confirmed to be a risk factor of mortality from MCD. Our study supports the association between social deprivation and MCD.

## Introduction

Meningococcal disease (MCD) is seen predominantly in infancy and early childhood, with a higher incidence in males [1]. As early as 1919, Rolleston [2] stated that "half the total cases occur in first five years of life", a statement that still applies today [3]. A significant change in age distribution was first observed in 1995, with a higher proportion of cases in older children and adults. There is now a second definite peak occurring in teenage years. The case fatality rates are high in under fives, low in school age children, and highest in those aged 25 years or more [4], [5].

In the United Kingdom, case fatality rates have been falling steadily since 1985, a trend which has been attributed to more efficient ascertainment of milder surviving cases [4], improvements in clinical management [6], [7], and the introduction of the meningococcal C conjugate vaccine [8]. The overall European picture has been similar to that in the United Kingdom, with overall case-fatality rates falling and the incidence of serogroup C disease also falling [9], [10], [11].

The association between incidence of MCD and socio-economic status is well described, with a relative risk of approximately two when comparing extremes of deprivation quintiles [12], [13], [14]. Mortality rates from MCD are also associated with social deprivation, with rates being increased in the most deprived children [12], [15].

The aims of the study were to describe the epidemiology of MCD in admissions to a single tertiary children's hospital on Merseyside, England over a 31 year period and to determine if there was any evidence of an association between MCD and socioeconomic variables within a well-defined geographic region.

## Methods

The data were collected prospectively (n = 692), (1988–1990, 1992–1994, 1997–1999, 2000–2002 , 2004–2006 and 2007–2009) and retrospectively (n = 465), in children admitted to Alder Hey Children's NHS Foundation Trust (formerly Royal Liverpool Children's Hospital) and Myrtle Street Hospital (until its closure in 1989). During the periods when the data were collected prospectively, a dedicated clinical research fellow was contacted each time a child with suspected MCD was admitted either to the emergency department or the intensive care unit. Detailed clinical, demographic, microbiology and laboratory data were collected on all children admitted prospectively. For cases collected retrospectively, a combination of hospital records, hospital electronic information system data and microbiology results from the Health Protection Agency (formerly Public Health Laboratory Service) were used.

Children were included in the database if they had microbiological confirmation or a probable diagnosis of MCD. **Microbiological confirmation** was defined as: positive cultures, positive polymerase chain reaction (PCR) for meningococcal DNA, detection of Gram-negative diplococci in CSF, or detection of meningococcal antigen in blood, CSF or synovial fluid; or abnormal CSF parameters (pleocytsis, low glucose, high protein) with positive serology for meningococcal infection; or positive throat swab in an ill child with fever and a petechial or purpuric rash. A **probable diagnosis** was made in children with negative microbiology, and who were either admitted to PICU (Paediatric Intensive Care Unit) with septicaemic shock and a purpuric/haemorrhagic rash, or who had post mortem findings consistent with a diagnosis of fulminant MCD [16].

The dataset contained information on date of admission, postcode of residence, gender, age, diagnosis (Meningococcal septicaemia alone (MS), Meningococcal Meningitis alone (MM) or a mixed picture (MM/MS)), Serogroup (B, C or Other), whether or not the patient survived and Glasgow Meningococcal Septicaemia Prognostic Score (GMSPS). Postcodes were used to generate the Townsend Score, a measure of social deprivation for the home address of each patient [17]. Using the online tool GeoConvert http://geoconvert.mimas.ac.uk/, we used the patient's postcodes to obtain coordinates for each of their residences, and to calculate their distance from the admitting hospital, Alder Hey Children's NHS Foundation Trust.

The Townsend score [17] is an area level measure of material deprivation, based on four indicators which are measured during the national census (i.e. the percentage of economically active residents who are unemployed, the percentage of private households who do not possess a car, the percentage of private households not owner occupied, and the percentage of private households with more than one person per room in a particular area). As it is a standardised score, a reference population needs to be selected. Using data from the 1981, 1991 and 2001 censuses, the four indicators were obtained for each census area within which patients admitted to Alder Hey Children's NHS Foundation Trust with meningococcal disease resided. The Townsend scores for each small area were then calculated, using Merseyside as the reference population. The patient was then assigned the Townsend score of their area of residence, for their decade of admission. For example, if a patient was admitted in 1994, they were assigned the Townsend score calculated for their area of residence using the 1991 census data. There were four missing Townsend scores, due to missing postcode information. There were 610 (52.7%) missing GMSPS scores, with the majority of missing scores occurring pre-1988, 1995–1997, and 2002–2004.

For the purpose of tabulating the data, four admission year groups were defined as 1977–1984, 1985–1992, 1993–2000 and 2001–2007. The third admission year group is the period of time in which a great increase in MCD cases was observed nationally, resulting in the introduction of the Meningococcal C vaccine in November 1999. Age groups were chosen in accordance with those used by the Health Protection Agency (Under 1 year, 1–4 years, 5–9 years, 10–14 years, over 15 years) [18].

The analysis was undertaken using R version 2.10.1 (www.r-project.org). Logistic regression modelling was used to assess the effect of covariates on risk of death. A backwards stepwise procedure was used for model selection.

Ethical approval was granted by Local Research Ethics Committee. Each prospective study was formally approved by the Local Research Ethics Committee at the time. Written consent was supplied by the parent/guardian of the patient using standardised consent forms for the institution, and gives consent for data collected during the study to be accessed and analysed by individuals for the purposes of research.

This is a single centre study run out of what was originally the Royal Liverpool Children's NHS Hospital. In 2009, the Trust received Foundation Trust status and the name changed to Alder Hey Children's NHS Foundation Trust. This is a purely administrative change in name. The protocol required review by one ethics committee at this institution. This protocol has received Institutional Review Board consideration throughout the duration of the study life. The hospital as an organization is the same and all ethical considerations have been complied with via the hospital's research review policies and standard operating procedures.

## Results

A total of 1157 patients were admitted to Alder Hey Children's NHS Foundation Trust with MCD during the 31 year period from 1977–2007 inclusive. The age range of patients admitted to Alder Hey was 0–16 years inclusive, although the dataset only contains one patient aged 16. Of the 1157 admissions, 43.5% were female and 56.5% were male.

**Figure 1:**
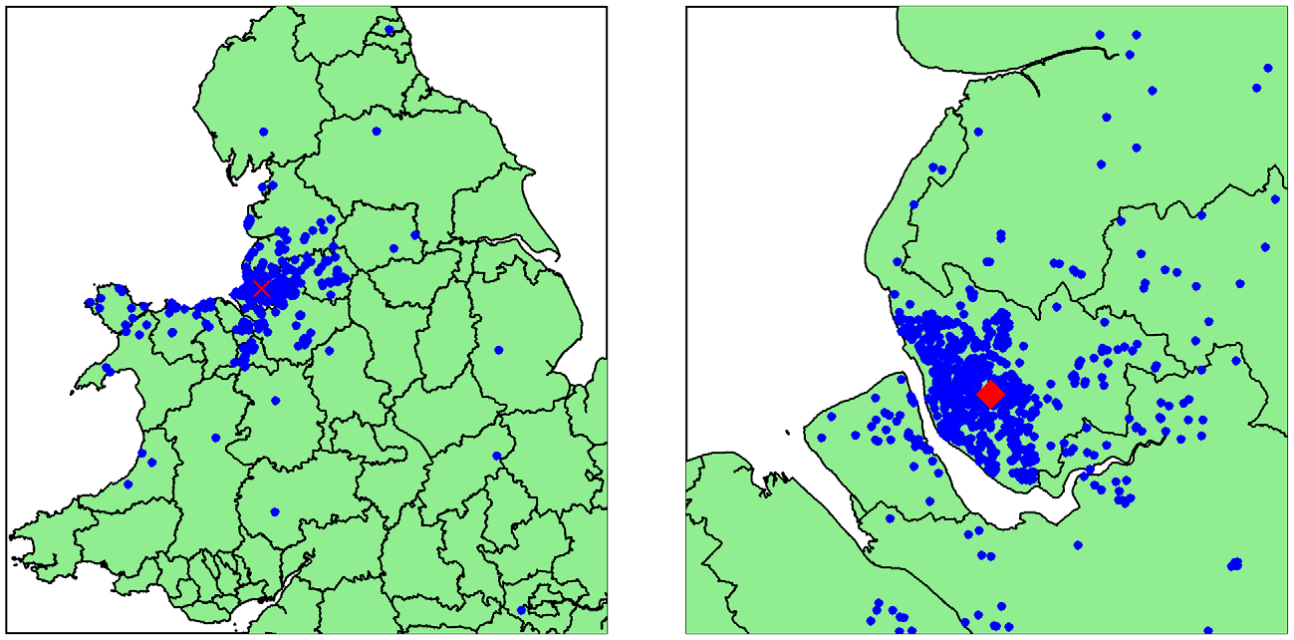
Location of MCD cases admitted to Alder Hey 1977–2007. Maps are of the UK, and of Merseyside only. The location of Alder Hey is clearly highlighted.

**Figure 2:**
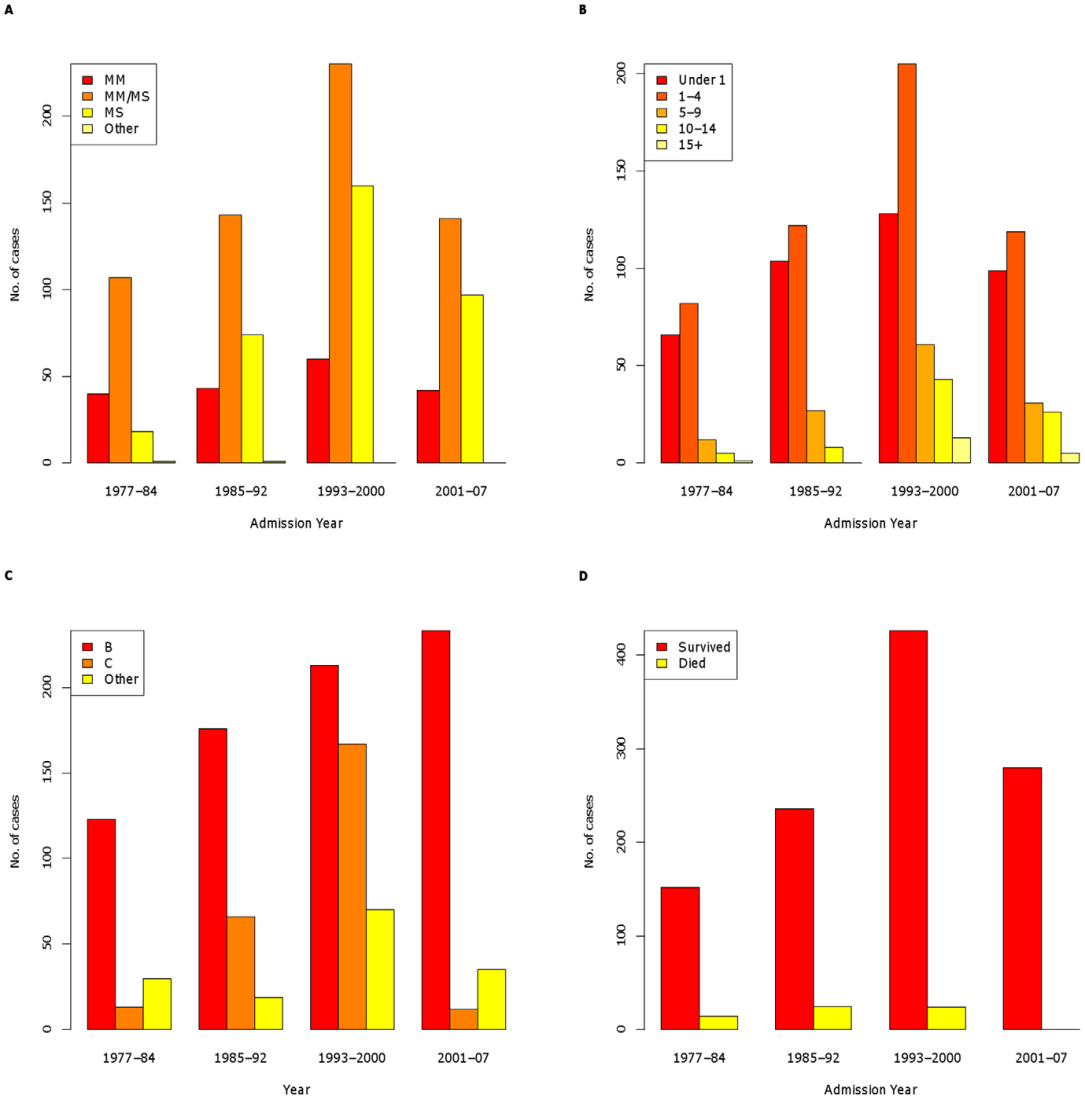
Histograms of numbers of cases by year group of admission. Cases are stratified by serogroup (A), age group (B), disease type (C) and outcome (D).

Figure 1 illustrates the geographical spread of postcode of residents of patients in the dataset. The majority of these are from the Merseyside area (864 patients – 74.7%), with some patients being resident in other areas of England and Wales, who were transferred in from other hospitals for intensive care management. The histograms in Figure 2 show the numbers of cases admitted to Alder Hey by period of admission, stratified by serogroup, age, disease type and outcome. Overall, there is a stepwise rise in cases between 1985–1992 and 1993–2000, after which the number of cases declines, following the introduction of the meningococcal C vaccine in 1999.

Throughout the study period, serogroup B has been the most common serogroup (64.4% overall) (Table 1). The percentage of serogroup C cases increased greatly during the middle two admission periods, and decreased significantly during the final admission period (Figure 2a) to the extent that no serogroup C cases occur after 2003.

**Table 1:** Alder Hey meningococcal disease cases by serogroup, age group and admission year group.

		Admission Year									
Age group	Serotype	1977–84		1985–92		1993–2000		2001–07		Total	
Under 1	B	52	(78.79%)	74	(71.15%)	71	(55.47%)	80	(80.81%)	277	(69.77%)
	C	5	(7.58%)	22	(21.15%)	38	(29.69%)	5	(5.05%)	70	(17.63%)
	OTHER	9	(13.64%)	8	(7.69%)	19	(14.84%)	14	(14.14%)	50	(12.59%)
1–4	B	56	(68.29%)	86	(70.49%)	93	(45.37%)	102	(85.71%)	337	(63.83%)
	C	7	(8.54%)	29	(23.77%)	85	(41.46%)	2	(1.68%)	123	(23.30%)
	OTHER	19	(23.17%)	7	(5.74%)	27	(13.17%)	15	(12.61%)	68	(12.88%)
5–9	B	10	(83.33%)	12	(44.44%)	30	(49.18%)	26	(83.87%)	78	(59.54%)
	C	1	(8.33%)	11	(40.74%)	18	(29.51%)	2	(6.45%)	32	(24.43%)
	OTHER	1	(8.33%)	4	(14.81%)	13	(21.31%)	3	(9.68%)	21	(16.03%)
10–14	B	4	(80.00%)	4	(50.00%)	16	(37.21%)	20	(76.92%)	44	(53.66%)
	C	0	(0.00%)	4	(50.00%)	18	(41.86%)	3	(11.54%)	25	(30.49%)
	OTHER	1	(20.00%)	0	(0.00%)	9	(20.93%)	3	(11.54%)	13	(15.85%)
15+	B	1	(100.00%)	0	(-)	3	(23.08%)	5	(100.00%)	9	(47.37%)
	C	0	(0.00%)	0	(-)	8	(61.54%)	0	(0.00%)	8	(42.11%)
	OTHER	0	(0.00%)	0	(-)	2	(15.38%)	0	(0.00%)	2	(10.53%)
All ages	B	123	(74.10%)	176	(67.43%)	213	(47.33%)	233	(83.21%)	745	(64.39%)
	C	13	(7.83%)	66	(25.29%)	167	(37.11%)	12	(4.29%)	258	(22.30%)
	OTHER	30	(18.07%)	19	(7.28%)	70	(15.56%)	35	(12.50%)	154	(13.31%)
Total		166		261		450		280		1157	

The majority of cases in each admission period were aged 4 and under (80% overall) (Table 2). There appears to have been a reduction in the proportion of cases in this age group between the first two time periods from 87.6% (374/427) to 74.1% (541/730) in the last two time periods. In contrast, there is an increase in the proportion of cases in the 10–14 age group between the first two time periods (13/427) and the last two time periods (69/730) (Table 2, Figure 2b). The differences between each of these pairs of proportions are statistically significant, with p-values less than 0.0001. However, in terms of absolute numbers, in comparing the periods 1985–1992 and 2001–2007 it was noted that there was no real reduction in the absolute number of cases under 4, with the number of cases being 226 and 218 respectively.

**Table 2:** Alder Hey meningococcal disease cases by age group and admission year group.

	Admission Year									
Age Group	1977–84		1985–92		1993–2000		2001–07		Total	
Under 1	66	(39.76%)	104	(39.85%)	128	(28.44%)	99	(35.36%)	397	(34.31%)
1–4	82	(49.40%)	122	(46.74%)	205	(45.56%)	119	(42.50%)	528	(45.64%)
5–9	12	(7.23%)	27	(10.34%)	61	(13.56%)	31	(11.07%)	131	(11.32%)
10–14	5	(3.01%)	8	(3.07%)	43	(9.56%)	26	(9.29%)	82	(7.09%)
15+	1	(0.60%)	0	(0.00%)	13	(2.89%)	5	(1.79%)	19	(1.64%)
Total	166		261		450		280		1157	

**Figure 3:**
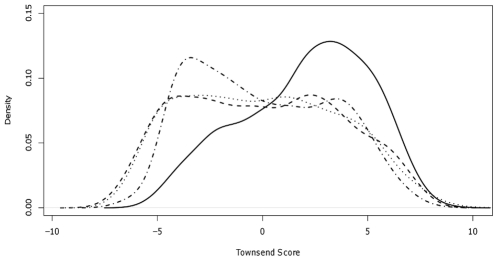
Smoothed density plot of Townsend scores for the reference population of Merseyside and MCD cases. The reference population is plotted for 1981 (dashed line), 1991 (dotted line), 2001 (dashed-dotted line). Townsend scores for the cases are represented by a solid line.

Density plots of the Townsend score for both the reference population of Merseyside by census year (1981, 1991 and 2001) and the cases (Figure 3) show that in general, the Townsend score of the cases is larger than that of the reference population. The mean Townsend score for the cases was 1.25, with a 95% confidence interval of (1.09, 1.41).

The disease profile changed over the study period. Clinical presentation with MS became more common with time, accounting for 34.6% in the last quarter, compared to 10.8% in the first (Table 3, Figure 2c). Accordingly, the proportion of cases presenting with MM reduced, though a mixed picture of MM/MS was consistently the most common presentation overall (53.7% in total).

**Table 3:** Alder Hey meningococcal disease cases by diagnosis and admission year group.

	Admission Year									
Diagnosis	1977–84		1985–92		1993–2000		2001–07		Total	
MM	40	(24.10%)	43	(16.48%)	60	(13.33%)	42	(15.00%)	185	(15.99%)
MM/MS	107	(64.46%)	143	(54.79%)	230	(51.11%)	141	(50.36%)	621	(53.67%)
MS	18	(10.84%)	74	(28.35%)	160	(35.56%)	97	(34.64%)	349	(30.16%)
OTHER	5	(0.60%)	1	(0.38%)	0	(0.00%)	0	(0.00%)	2	(0.17%)
Total	166		261		450		280		1157	

Overall the proportion of deaths for the entire study period was 5.5% (63 deaths). The proportion of deaths was highest during the 1985–1992 admission period (9.6%), and subsequently reduced significantly, such that there were no deaths in the study population after 2000 (Table 4 and Figure 2d). Overall, the proportion of deaths was highest in the MS group (7.7%) compared to the MM/MS group (5.2%) and the MM group (2.2%) (Table 4). There were statistically significant differences amongst these proportions (p = 0.0233). Of the patients with serogroup B disease, 4.4% died (33/745) compared to 9.3% of children with serogroup C infection (24/258) (p = 0.0058). In absolute terms, most of the deaths occurred in infants (30.2%) and the 1–4 years age group (47.6%). The overall proportion of deaths in infants was 4.8% (19/397), 5.7% in the 1–4 years age group, 3.8% (5/131) in the 5–9 years group, 7.3% (6/82) in the 10–14 years group and 15.8% (3/19) in the over 15 years age group.

**Table 4:** Number and percentage of deaths by diagnosis and admission year group.

	Admission Year									
Diagnosis	1977–84		1985–92		1993–2000		2001–07		Total	
MM	0	(0.00%)	0	(0.00%)	4	(6.67%)	0	(0.00%)	4/185	(2.17%)
MM/MS	10	(9.35%)	13	(9.09%)	9	(3.91%)	0	(0.00%)	32/621	(5.15%)
MS	4	(22.22%)	12	(16.22%)	11	(6.88%)	0	(0.00%)	27/349	(7.74%)
OTHER	0	(0.00%)	0	(0.00%)	-	-	-	-	0/2	(0.00%)
Total	14/166	(8.43%)	25/261	(9.58%)	24/450	(5.33%)	0/280		63/1157	(5.45%)

Table 5 shows the results of fitting the multiple logistic model to the data, thereby exploring which factors are associated with risk of death in patients admitted to Alder Hey. The final model included disease presentation and serogroup. Adding any or all of age, sex, Townsend score and distance from Alder Hey gave no significant improvement in fit over the final model. The risk of death was higher for patients with MS and infection with serogroup C disease. The odds ratio (OR) for death was 3.5 (95% CI 1.18 to 10.08) in the MS group, compared to MM. The OR for death in the serogroup C group, compared to serogroup B was 2.18 (95% CI 1.26 to 3.80).

**Table 5:** Multiple logistic regression model for risk of death.

	Log(OR)	OR	CI	p-value
Intercept	-3.9928	0.0184	(0.006669, 0.051035)	<<0.01
Diagnosis:MM/MS	0.7824	2.3832	(0.828198, 6.858011)	0.11
Diagnosis: MS	-0.2853	3.4537	(1.183001, 10.082928)	0.02
Serogroup: C	0.8685	2.1867	(1.258814, 3.798563)	0.01
Serogroup: Other	1.2394	0.7518	(0.287441, 1.966337)	0.56

## Discussion

We undertook a descriptive epidemiological study of 1157 children admitted to Alder Hey Children's NHS Foundation Trust with MCD during the 31 year period from 1977–2007. This is the largest published dataset containing clinical, microbiological and socioeconomic data of children with MCD from a single region. Merseyside has a higher incidence of MCD than the rest of the North West of England, and England and Wales as a whole. Data from the Health Protection Agency indicate that the incidence of MCD in England and Wales for 2008 and 2009 was 2.26/100,000 and 1.88/100,000 respectively. By comparison, the figures for the North West of England and Merseyside respectively were: 2008: 2.39 and 2.79/100,000 and 2009: 2.35 and 3.63/100,000.

There was a dramatic increase in the number of cases between 1995 and 1998, after which the number declined, following the introduction of the meningococcal C vaccine. The success of the meningococcal C vaccine is evident in the fact that there are no serogroup C cases after 2003, despite a significant rise in the proportion of serogoup C cases in the period preceding this. In terms of demographic characteristics, we observed a slight male bias in presentations from the Merseyside area. The cases appear to be drawn from a relatively more deprived population than the Merseyside reference population. A total of 80% of cases were in the 1–4 year age group, but over the data collection period there is a relative increase in the proportion of cases in older age groups. Molecular diagnosis for detection of meningococcal DNA was introduced in 1996. As a result, ascertainment increased [19], [20], and this increased ascertainment most likely explains the observation that absolute numbers of under 4yr olds has been fairly constant between the periods 1985–1992 and 2001–2007. Specifically, with a relative decrease in the proportion of cases in this age group, and fewer cases overall, one would expect a decrease in absolute numbers. However increased case ascertainment with meningococcal PCR is the most likely explanation for why this is not observed.

Presentation with a mixed picture of MM/MS was consistently the most common clinical presentation overall, though MS became a relatively more common presentation over the data collection period, rising from 11% to 35%. This increase in proportion of cases with MS is likely to be due to less lumbar punctures being performed, and in particular the recommendation that lumbar puncture is not performed in children with a purpuric rash. Hence, cases that would have previously been ascribed to MM/MS following a lumbar puncture are now more likely to be ascribed to MS. The low fatality rate despite higher proportions of septicaemia in the last study period (0/97) is in all likelihood due to improved mortality as a result of prompt recognition and aggressive management of the disease [6], [7]. Mortality was greatest during the 1985–1992 admission period (9.6%), but subsequently there was a significant reduction. There were no deaths between 2001 and 2007. This significant reduction in mortality from MCD has been attributed to earlier recognition of disease, and more aggressive management of cases on admission to hospital and in intensive care [6], [7].

MS (compared to MM) and serogroup C (compared to serogroup B) emerged as independent risk factors for mortality. MS is the most fulminant disease presentation of MCD as it can rapidly progress to septic shock, multi-organ failure and death. Prompt recognition and early aggressive management of the critically ill child has led to significant improvements in outcome in children with meningococcal septic shock [21]. Social deprivation and distance from hospital were not significant risk factors for MCD mortality in our analysis.

By examining this unique dataset, containing data which has been collected over a 31 year period from 1977-2007, we have been able to describe a range of socio-demographic and clinical characteristics, and explore risk factors for mortality. The strengths of the study are the large dataset from a single centre, the fact that most of the data was collected prospectively by a dedicated clinical research fellow, and that data on clinical presentation in addition to microbiology and postcode were collected. Weaknesses of this study include the fact that the analysis is linked to a single centre, for which it is not possible to define a discrete catchment population. Thus we were unable to undertake an area-based study of incidence of MCD and were limited to a largely descriptive analysis. Further, there is potential for ascertainment bias with respect to information on patient mortality as the mortality data only takes into account children who reach this tertiary centre. In exploring the effect of deprivation, we used the population of Merseyside as the reference population, and generated Townsend scores for successive time periods. It was not possible to use more current deprivation measures such as Index of Multiple Deprivation [22], as this was only available from 2000 onwards. We were limited to cross-sectional analysis by deprivation, since the Townsend score is a relative measure, and cannot be used as an absolute measure of deprivation to identify absolute change over time. We have used a small-area-based measure of deprivation, since we did not have access to individual-level measures of socio-economic status, and any associations are thus ecological [23].

In our analysis we found that the cases admitted to Alder Hey were from a relatively more deprived population than the Merseyside reference population, but that deprivation score was not a risk factor for mortality. This is in line with other studies in Wales and the Eastern region of England that have shown an association between MCD and social deprivation at an area level [13], [14]. Unlike our study, Heyderman et al. [12] have suggested a link between social deprivation and meningococcal mortality, possibly mediated by factors such as overcrowding, damp living conditions, environmental tobacco smoke exposure and viral upper respiratory tract infections, which may lead to increased exposure and/or carriage of Neisseria meningitidis or impaired host defences against bacterial invasion.

Olowokure et al. [24] demonstrate an interaction between urban/rural indicators and social deprivation, with the most deprived community in the urban areas being twice as likely as the most deprived community in the rural area to have MCD. In our study, most cases were classified as living in urban areas, therefore we were not able to demonstrate an association with rural or urban residence. However, distance from hospital did not have a significant effect on risk of mortality from MCD.

In conclusion, our large dataset demonstrates that most cases occur in the first five years of life, and confirms MS and serogroup C disease as independent risk factors for mortality. Additionally, the cases admitted to Alder Hey were from a relatively more deprived population than the Merseyside reference population, but deprivation score was not a risk factor for mortality.
